# Diagnostic accuracy of preoperative ultrasonography in predicting contralateral inguinal hernia in children: a systematic review and meta-analysis

**DOI:** 10.1007/s00330-018-5625-6

**Published:** 2018-07-27

**Authors:** K. M. A. Dreuning, C. E. M. ten Broeke, J. W. R. Twisk, S. G. F. Robben, R. R. van Rijn, J. I. M. L. Verbeke, L. W. E. van Heurn, J. P. M. Derikx

**Affiliations:** 1 Department of Paediatric Surgery, Paediatric Surgical Center of Amsterdam, Emma Children’s Hospital AMC & VU University Medical Center, Meibergdreef 9, 1105 AZ Amsterdam, The Netherlands; 2 Department of Methodology and Applied Biostatistics, and the Amsterdam Public Health Research Institute, VU University Medical Center, De Boelelaan 1089a, 1081 HV Amsterdam, The Netherlands; 3 Department of Radiology, Maastricht University Medical Center, P. Debyelaan 25, 6229 HX Maastricht, The Netherlands; 4 Department of Radiology, Academic Medical Center/Emma Children’s Hospital, Meibergdreef 9, 1105 AZ Amsterdam, The Netherlands; 5 Department of Radiology and Nuclear Medicine, VU University Medical Center, De Boelelaan 1117, 1081 HV Amsterdam, The Netherlands

**Keywords:** Hernia, inguinal, Ultrasonography, Child

## Abstract

**Objectives:**

The incidence of children developing metachronous contralateral inguinal hernia (MCIH) is 7–15%. Contralateral groin exploration during unilateral hernia repair can prevent MCIH development and subsequent second surgery and anaesthesia. Preoperative ultrasonography is a less invasive strategy and potentially able to detect contralateral patent processus vaginalis (CPPV) prior to MCIH development.

**Methods:**

We queried MEDLINE, Embase and Cochrane library to identify studies regarding children aged < 18 years diagnosed with unilateral inguinal hernia without clinical signs of contralateral hernia, who underwent preoperative ultrasonography of the contralateral groin. We assessed heterogeneity and used a random-effects model to obtain pooled estimates of sensitivity, specificity and area under the receiver operating characteristic curve (AUC).

**Results:**

Fourteen studies (2120 patients) were included, seven (1013 patients) in the meta-analysis. In studies using surgical exploration as reference test (*n* = 4, 494 patients), pooled sensitivity and specificity were 93% and 88% respectively. In studies using contralateral exploration as reference test following positive and clinical follow-up after negative ultrasonographic test results (*n* = 3, 519 patients), pooled sensitivity was 86% and specificity 98%. The AUC (0.984) shows high diagnostic accuracy of preoperative ultrasonography for detecting CPPV, although diagnostic ultrasonographic criteria largely differ and large heterogeneity exists. Reported inguinal canal diameters in children with CPPV were 2.70 ± 1.17 mm, 6.8 ± 1.3 mm and 9.0 ± 1.9 mm.

**Conclusion:**

Diagnostic accuracy of preoperative ultrasonography to detect CPPV seems promising, though may result in an overestimation of MCIH prevalence, since CPPV does not invariably lead to MCIH. Unequivocal ultrasonographic criteria are mandatory for proper diagnosis of CPPV and subsequent prediction of MCIH.

**Key Points:**

*• Diagnostic accuracy of preoperative ultrasonography for detection of CPPV in children with unilateral inguinal hernia is high.*

*• Preoperative ultrasonographic evaluation of the contralateral groin assumedly results in an overestimation of MCIH prevalence.*

*• Unequivocal ultrasonographic criteria are mandatory for proper diagnosis of CPPV and risk factor identification is needed to predict whether CPPV develops into clinically apparent MCIH.*

**Electronic supplementary material:**

The online version of this article (10.1007/s00330-018-5625-6) contains supplementary material, which is available to authorized users.

## Introduction

Inguinal hernia is the most common surgical condition in childhood and presents in approximately 0.8–5% of term children and more than 30% in premature born children [1, 2]. At the time of surgery, the hernia is unilateral in 80% of children. However, 7–15% of children develop a hernia on the opposite side after unilateral hernia repair, a metachronous contralateral inguinal hernia (MCIH), with the highest risk in children under the age of 6 months or with initial left-sided hernia [3, 4]. Medical history and physical examination of the groin are not adequate to detect a hidden (or asymptomatic) patent processus vaginalis (PV) [5–7], which is likely to develop into an MCIH. MCIH necessitates a second operation and anaesthesia. The repeated use of anaesthetics possibly affect the development of children’s brains [8]. Preventive strategies have been proposed since the 1950s and exploration of the contralateral groin during unilateral hernia repair is frequently used to detect contralateral patent processus vaginalis (CPPV). However, contralateral exploration carries risks of operative complications (e.g. wound infection, haematoma or testicular atrophy) and is unnecessary if the processus vaginalis is closed [9, 10]. Therefore, management of the contralateral groin remains controversial.

If contralateral hernia development could be predicted prior to symptomatic hernia repair, individual recommendations regarding contralateral exploration can be made more consciously. Accurate preoperative evaluation of the contralateral inguinal canal could prevent unnecessary contralateral exploration and might reduce the incidence of children developing MCIH and obviating the need for a second operation and anaesthesia.

Previous studies show that preoperative ultrasonography has high accuracy for detecting both symptomatic and asymptomatic inguinal hernias in children, as the sensitivity for diagnosing CPPV is 98% and 97%, respectively [6, 11, 12]. False negative rates of ultrasonography-based detection of CPPV are difficult to determine since most studies do not evaluate the contralateral inguinal canal intra- or postoperatively [13].

The aim of this systematic review and meta-analysis is to assess the diagnostic accuracy of preoperative ultrasonography of the contralateral groin to detect CPPV and its potential value to predict development of MCIH in children with unilateral inguinal hernia.

## Methods

### Protocol

A systematic review was conducted in accordance with the Preferred Reported Items for Systematic Reviews and Meta-Analysis (PRISMA) statement on all literature regarding preoperative ultrasonography as a diagnostic tool for contralateral inguinal hernia in children. The protocol was registered (PROSPERO 2017, CRD42017058269).

### Literature search

We queried EMBASE, MEDLINE, PubMed, the Cochrane library databases and reference lists of eligible articles for the selection of studies. The search strategy was conducted in December 2016 (Appendix 1) and updated in November 2017.

### Eligibility criteria

Articles were considered eligible irrespective of language or publication date and status. Inclusion criteria were (a) children under 18 years old diagnosed with unilateral inguinal hernia who had (b) no clinical signs of contralateral hernia and (c) underwent preoperative ultrasonography of the contralateral groin prior to surgical hernia repair. Patients diagnosed with (a) bilateral inguinal hernia or (b) inguinal hernia associated with non-descendent testis were excluded from data analysis.

### Study selection and methodological quality assessment

Two reviewers independently performed the screening and selection of studies based on title and abstract, and full text for final selection. The full text of articles was retrieved by contacting the authors if it was not available via our library and attempts were made to translate articles that were not reported in English. The most complete study was included if the same data was repeatedly reported. Modified Quality Assessment of Diagnostic Accuracy Studies (QUADAS-2) tool was used to independently assess the methodological quality of the included studies. Inconsistencies were solved by second joint review of the literature or by consulting a third independent review author.

### Data extraction and analysis

Appendix 2 comprises data that was systematically extracted from the studies included in this systematic review by the two reviewers and recorded in a data collection form. Missing data were calculated if possible and unpublished data or further details were retrieved by contacting study author(s).

Surgery, i.e. contralateral exploration, and clinical follow-up were used as the reference standard following positive index test results. Negative index test results were followed by surgery, clinical follow-up or no reference test. Studies that performed contralateral exploration in all patients, regardless of the index test results, are referred to as ‘complete cases’. Studies that solely performed contralateral exploration following positive index test results are referred to as ‘incomplete cases’.

### Statistical analysis

Statistical analysis was performed using Review Manager (version 5.3.5) and Meta-DiSc (version 1.4). The available data were inserted in 2 × 2 tables to compute sensitivity, specificity, positive predictive value (PPV) and negative predictive value (NPV) for each study. A random-effects model was used to obtain pooled estimates of sensitivity and specificity including their 95% confidence intervals. Heterogeneity between studies was assessed using the *χ*^2^ and *I*^2^ statistic. The summary receiver operating characteristic (SROC) curve was used to estimate the area under the curve (AUC) that represented overall diagnostic performance of the index test.

## Results

### Literature search

**Fig. 1:**
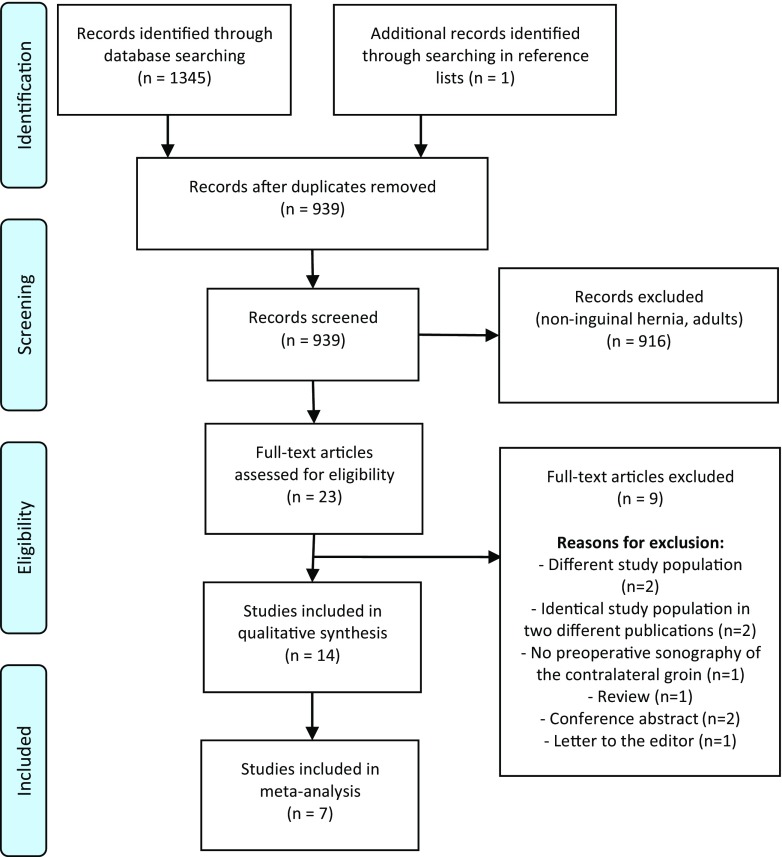
PRISMA flow chart of the study selection

Literature search and manual reference analysis yielded 1346 potentially eligible studies. Duplicates were removed and 916 records were excluded after the initial screening. Nine more records were excluded after full text screening and 14 studies were included in this review (Fig. 1).

### Study characteristics

The 14 studies were published between 1992 and 2017. They included 2120 children diagnosed with unilateral inguinal hernia undergoing preoperative ultrasonography of the contralateral groin. Study and index test characteristics are described in Tables 1 and 2. If preoperative ultrasonography yielded positive test results, contralateral exploration (*n* = 12 studies) and clinical follow-up (*n* = 2 studies) were performed, negative index test results were followed by contralateral exploration (*n* = 4 studies), clinical follow-up (*n* = 7 studies) or no other reference test (*n* = 3 studies). Generally, the index test (preoperative ultrasonography) was considered positive if there was flow of abdominal fluid or structures through an open PV or the diameter of the PV was more than 4 mm; it was considered negative if the PV was closed or the diameter was less than 4 mm. The flow chart in Appendix 3 shows how diagnostic test accuracy results were determined. Seven studies were included in the meta-analysis [14–20].

**Table 1:** Study characteristics and patient demographics of the included studies (*n* = 14)

Author	Year	Country	Study design	Index test	Reference test		Patients *n*	Male *n* (%)	Female *n* (%)	Age (mean/median)	Follow-up (months)
					Positive ITR	Negative ITR					
Chen	1998	Taiwan	Cohort study	Ultrasound	Surgical exploration	None	203	203 (100)	–	3 days–13 years (mean 32.6 months)	–
Chou	1996	Taiwan	Cohort study	Ultrasound	Surgical exploration	Surgical exploration	179	Unclear	Unclear	3 days–14 years (mean 43.1 months)	–
Erez	1996	Israel	Cohort study	Ultrasound	Surgical exploration	Clinical follow-up	200	Unclear	Unclear	6 months–13 years	36–48
Hasanuzzaman	2011	Bangladesh	Cohort study	Ultrasound	Surgical exploration	None	30	17 (56.7)	13 (43.3)	2.5–14 years	–
Hata	2004	Japan	Cohort study	Ultrasound	Surgical exploration	None	348	168 (48.3)	180 (51.7)	Unclear	–
Kaneda	2015	Japan	Cohort study	Ultrasound	Clinical follow-up	Clinical follow-up	105	47 (44.8)	58 (55.2)	5 days–13 years (median 47 months)	36
Kazez	1998	Turkey	Cohort study	Ultrasound	Surgical exploration	Surgical exploration	46	41 (89.1)	5 (10.9)	35 days–2 years (mean 11 months)	–
Kazez	2001	Turkey	Cohort study	Ultrasound	Clinical follow-up	Clinical follow-up	29	29 (100)	–	50 days–4 years (mean 20.4 months)	11
Kervancioglu	2000	Turkey	Cohort study	Ultrasound	Surgical exploration	Clinical follow-up	121	Unclear	Unclear	36 day–15 years (mean 4.7 years)	6–12
Lawrenz	1994	Scotland	Cohort study	Ultrasound	Surgical exploration	Surgical Exploration	23	23 (100)	–	0–325 days (mean 86 days)	–
Shehata	2013	Saudi Arabia	Cohort study	Ultrasound	Surgical exploration	Surgical Exploration	246	207 (84.1)	39 (15.9)	0–1 year (median 45 weeks)	–
Toki	2003	Japan	Cohort study	Ultrasound	Surgical exploration	Clinical follow-up	271	163 (60.1)	108 (39.9)	0–14 years (median 2 years)	24
Uno	1992	Japan	Case-control study	Ultrasound	Surgical exploration	Clinical follow-up	32	21 (65.6)	11 (34.4)	< 15 years	≥ 12
Zaidi	2017	Pakistan	Cross-sectional study	Ultrasound	Surgical exploration	Clinical follow-up	287	264 (92.0)	23 (8.0)	0.5–132 months	≤ 24

*ITR* index test results

**Table 2:** Index test characteristics and diagnostic test criteria of the included studies (*n* = 14)

Author, year	Index test	Transducer	Examination	Diagnostic criteria for contralateral PPV or inguinal hernia	WLIR^a^ of contralateral hernia
Chen, 1998	Ultrasound	7.0 MHz linear array transducer	At rest and straining	(A) Presence of bowel loops or omentum in inguinal canal (B) Presence of fluid in processus vaginalis (C) No bowel loops, omentum or fluid in processus vaginalis, but widening of the cord at the level of internal ring. WLIR > 4 mm is considered an occult hernia	PPV > 4 mm
Chou, 1996	Ultrasound	7.0 MHz linear array transducer	At rest and straining	(A) Diameter of internal inguinal ring > 4 mm (B) Presence of fluid in the processus vaginalis (C) Presence of bowel loops or other peritoneal structures in the inguinal canal	PPV > 4 mm
Erez, 1996	Ultrasound	7.0 MHz linear array transducer	At rest, no straining	The presence of fluid, characterised by a homogeneous and more hypoechoic appearance, wider than a PPV	PPV > 3 mm (< 1 year) PPV > 4 mm (1–2 years) PPV > 5 mm (> 2 years)
Hasanuzzaman, 2011	Ultrasound	7.5 MHz linear array transducer	At rest and straining	CPPV that was not detectable at rest could be visualised as a hydrocele owing to the inflow or peritoneal fluid into a processus vaginalis on straining	Unclear
Hata, 2004	Ultrasound	7.5 MHz linear array transducer	At rest and straining	(A) Hydrocele, owing to inflow of physiologic ascites into the processus vaginalis, detectable while straining (B) Expanded processus vaginalis owing to the protrusion of a viscus, detectable while straining (C) Hydrocele > 10 mm in longitudinal length	Unclear
Kaneda, 2015	Ultrasound	10.0 MHz linear array transducer	At rest, no straining	Major axis of the contralateral PPV in millimetres	Unclear
Kazez, 1998	Ultrasound	5.0 MHz linear array transducer	Unclear	(A) PPV: 2–3 mm (B) Hernia: > 4 mm	PPV > 4 mm
Kazez, 2001	Ultrasound	7.5 MHz linear array transducer	Unclear	(A) PPV: 2–4 mm (B) Hernia > 4 mm	PPV > 4 mm
Kervancioglu, 2000	Ultrasound	7.5 MHz linear array transducer	At rest, no straining	(A) Presence of fluid, bowel loops or omentum in the inguinal canal (B) PPV width ≥ 4 mm (C) Extension of bowel loops or omentum into the scrotum	PPV > 4 mm
Lawrenz, 1994	Ultrasound	7.0 MHz linear array transducer	At rest and straining	(A) PPV (B) PPV and hydrocele	Unclear
Shehata, 2013	Ultrasound	10.0 MHz linear array transducer	At rest and straining	(A) PPV with intra-abdominal organ observed in inguinal canal (B) PPV seen as a cyst at the internal ring of the inguinal canal (C) PPV is widened with increases in abdominal pressure and length of the PPV > 20 mm	Unclear
Toki, 2003	Ultrasound	10.0 MHz annular array transducer	At rest and straining	(A) PPV with intra-abdominal organ observed in inguinal canal (B) PPV is cyst-like, exceeding 20 mm along the major axis at the internal ring of the inguinal canal (C) PPV is widened with abdominal pressure increment and length of the PPV ≥ 20 mm (D) PPV contains moving fluid without PPV widening	Unclear
Uno, 1992	Ultrasound	3.75 MHz convex array transducer	Unclear	Unclear	Boys ≥ 7 mm Girls ≥ 4 mm
Zaidi, 2017	Ultrasound	7.5–11.0 MHz linear array transducer	Unclear	Inguinal hernia/PPV if maximum observed diameter of the inguinal canal at the internal ring ≥ 4.5 mm	PPV ≥ 4.5 mm

*PPV* patent processus vaginalis, *WLIR* width of low echoic region of the internal ring

^a^WLIR of contralateral side as criteria for diagnosis of contralateral PPV or inguinal hernia

### Methodological quality

**Fig. 2:**
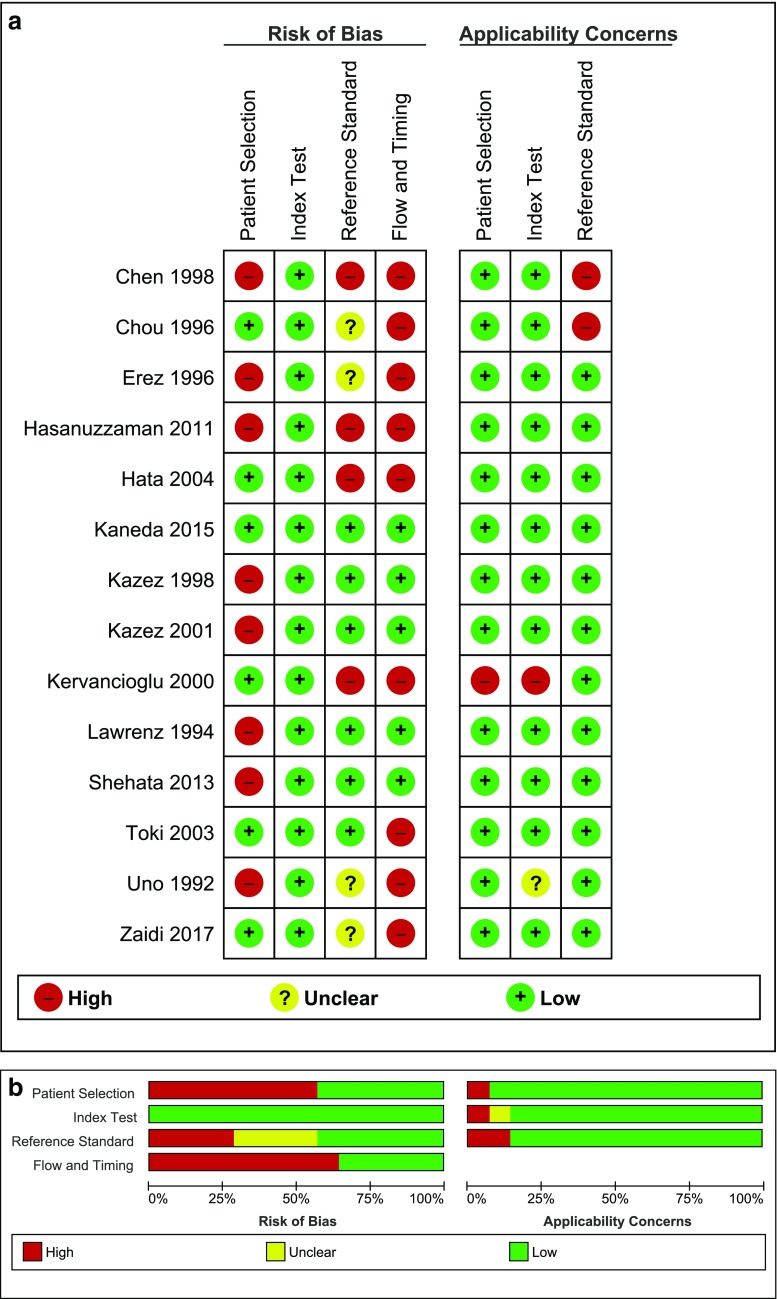
Methodological quality assessment of the included studies using the QUADAS-2 tool. **a** Risk of bias and applicability concerns summary about each domain are shown for each included study; **b** risk of bias and applicability concerns about each domain presented as percentages across included studies. Red circle with minus sign, high risk; yellow circle with question mark, unclear risk; green circle with plus sign, low risk

Quality assessment using the QUADAS-2 tool indicated a moderate to high risk of bias concerning the selection of patients, because among others patient selection was not specified [18] and exclusions were made based on sex [5, 16, 21] or age [15, 17, 22]. The risk of bias regarding the flow and timing domain was also considered high as most studies limited the use of contralateral exploration as the reference standard to a preset part of their study population [5, 7, 11, 18–20, 22, 23]. Assessment of bias regarding the index test domain and overall applicability concerns were considered low (Fig. 2a, b).

### Studies excluded from the meta-analysis

Seven studies were excluded from the meta-analysis. In five studies, diagnostic accuracy test results could not be determined because no reference test was performed [5, 11, 22] or the diagnostic or reference test results could not be extracted, calculated or obtained from the authors [7, 23] (Table 3). Two studies were excluded because clinical follow-up was used as reference standard in all children, irrespective of the index test results. Their diagnostic accuracy test results were therefore related to the occurrence of MCIH in children during clinical follow-up and could not be used to evaluate the diagnostic accuracy of preoperative ultrasonography to detect CPPV [13, 21].

**Table 3:** Diagnostic accuracy test results from studies that were excluded from the meta-analysis (*n* = 7)

Author, year	Patients (*n*)	TP	FP	FN	TN	Follow-up (months)	Sensitivity (%)	Specificity (%)	PPV (%)	NPV (%)	US CPPV/MCIH (%)	Clinical CPPV/MCIH (%)
Chen, 1998	203	62	3	–	–	–	–	–	95.4	–	32.0	–
Hasanuzzaman, 2011	30	11	1	–	–	–	–	–	91.7	–	40.0	–
Hata, 2004	348	74	4	–	–	–	–	–	94.9	–	22.4	–
Kaneda, 2015^a^	105	9	27	2	67	36	81.8	71.3	25	97.1	34.3	10.5
Kazez, 2001^a^	29	0	11	0	18	11	–	62	–	100	37.9	0
Kervancioglu, 2000	121	10	0	–	–	6–12	–	–	100	–	8.3	–
Toki, 2003	271	–	–	4	–	24	–	–	–	–	–	–

*TP* true positive, *FP* false positive, *FN* false negative, *TN* true negative, *PPV* positive predictive value, *NPV* negative predictive value, *US* ultrasound, *CPPV* contralateral patent processus vaginalis, *MCIH* metachronous contralateral inguinal hernia

^a^Clinical findings during follow-up were used to calculate the amount of true positives (TP), false positives (FP), false negatives (FN) and true negatives (TN)

### Diagnostic accuracy of preoperative ultrasonography in complete cases

**Fig. 3:**
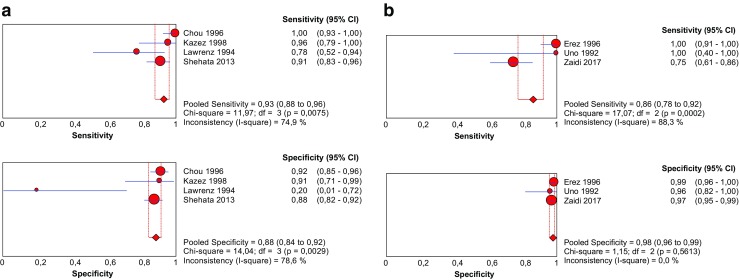
Pooled sensitivity and specificity forest plots including the 95% confidence intervals (CI) of **a** complete cases and **b** incomplete cases

**Fig. 4:**
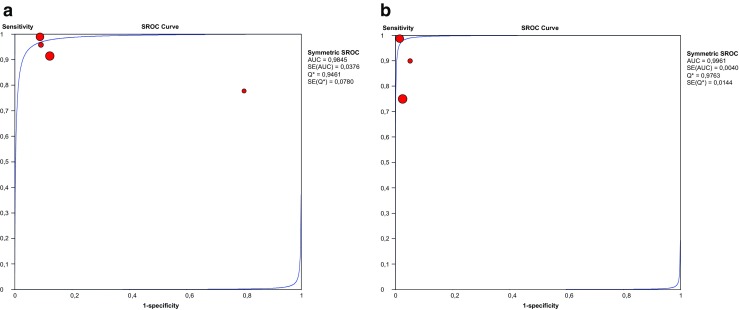
Summary receiver operating characteristic curve of preoperative ultrasonography for detection of contralateral patent processus vaginalis in **a** complete cases and **b** incomplete cases. SROC summary receiver operating characteristic curve, AUC area under the curve

A total of 494 children from four different studies were included as complete cases (Table 4). The diagnostic sensitivity ranged from 78% (at a specificity of 20%) to 100% (at a specificity of 92%) [14–17]. The pooled sensitivity was 93% (95% CI 88–96%; *χ*^2^ = 11.97, *p* < 0.01; *Ι*^2^ = 74.9%) and the pooled specificity was 88% (95% CI 84–92%; *χ*^2^=14.04, *p* < 0.01; *Ι*^2^ = 78.6%) (Fig. 3a). Significant heterogeneity exists in the estimated sensitivity (*Ι* ^2^ = 74.9%, *p* < 0.01) and specificity (*Ι*^2^ = 78.6%, *p* < 0.01) between studies. The AUC of the SROC was 0.984 (Fig. 4a).

**Table 4:** Diagnostic accuracy test results from complete cases included in the meta-analysis (*n* = 4). Complete cases: all patients underwent both the index test (preoperative ultrasonography) and reference test (surgical exploration), irrespective of the index test results. Clinical findings during follow-up (if reported) were used to calculate the amount of false negatives (FN) and true negatives (TN)

Author, year	Patients (*n*)	TP	FP	FN	TN	Sensitivity (%)	Specificity (%)	PPV (%)	NPV (%)	US CPPV^a^ (%)	OR CPPV^b^ (%)
Chou, 1996	179	49	11	0	119	100	91.5	81.7	100	33.5	27.4
Kazez, 1998	46	23	2	1	20	95.8	90.9	92	95.2	54.3	52.2
Lawrenz, 1994	23	14	4	4	1	77.8	20.0	77.8	20.0	78.3	78.3
Shehata, 2013	246	75	20	7	144	91.7	87.7	75.9	96.2	38.6	33.3

*TP* true positive, *FP* false positive, *FN* false negative, *TN* true negative, *PPV* positive predictive value, *NPV* negative predictive value, *US* ultrasound, *CPPV* contralateral patent processus vaginalis

^a^Amount (%) of contralateral patent processus vaginalis as determined by ultrasonography

^b^Amount (%) of contralateral patent processus vaginalis detected intraoperatively

### Diagnostic accuracy of preoperative ultrasonography in incomplete cases

Three studies with 519 children were included in this part of the meta-analysis (Table 5). The follow-up period ranged from 12 to 48 months. The diagnostic sensitivity ranged from 75% (at a specificity of 97%) to 100% (at a specificity of almost 99%) [13, 18–20]. The pooled sensitivity was 86% (95% CI 78–92%; *χ*^2^ = 17.07, *p* < 0.01; *Ι* ^2^ = 88.3%) and the pooled specificity was 98% (95% CI 96–99%; *χ*^2^ = 1.15, *p* < 0.01; *Ι*^2^ = 0.0%) (Fig. 3b). The AUC of the SROC was 0.996 (Fig. 4b).

**Table 5:** Diagnostic accuracy test results from incomplete cases included in the meta-analysis (*n* = 3). Incomplete cases: surgical exploration was only performed when the index test (preoperative ultrasonography) yielded positive test results. Clinical findings during follow-up (if reported) were used to calculate the amount of false negatives (FN) and true negatives (TN)

Author, year	Patients (*n*)	TP	FP	FN	TN	Follow-up (months)	Sensitivity (%)	Specificity (%)	PPV (%)	NPV (%)	US CPPV^a^ (%)	Clinical MCIH^b^ (%)
Erez, 1996	200	38	2	0	160	36–48	100	98.8	95.0	100	20.0	19.0
Uno, 1992	32	4	1	0	27	≥ 12	100	96.4	80	100	15.6	12.5
Zaidi, 2017	287	39	6	13	229	≤ 24	75.0	97.4	86.7	94.6	15.7	18.1

*TP* true positive, *FP* false positive, *FN* false negative, *TN* true negative, *PPV* positive predictive value, *NPV* negative predictive value, *US* ultrasound, *CPPV* contralateral patent processus vaginalis, *MCIH* metachronous contralateral inguinal hernia

^a^Amount (%) of contralateral patent processus vaginalis as determined by ultrasonography

^b^Amount (%) of metachronous contralateral inguinal hernias that occurred during clinical follow-up

### Diameter of the width of low echoic region of the internal ring (WLIR) [19]

Three studies including 424 children contributed data regarding the WLIR of the contralateral inguinal ring. In children without patency of the contralateral PV the mean diameter of the contralateral ring was 1.34 ± 1.26 mm, 3.5 ± 0.4 mm [13, 20] or less than 6.0 mm in boys in particular [19]. The mean WLIR in children diagnosed as having a CPPV was 6.8 ± 1.3 mm and in children diagnosed as having a contralateral hernia the mean WLIR was 2.70 ± 1.17 mm or 9.0 ± 1.9 mm [13, 20].

## Discussion

In this review, we aimed to assess the diagnostic accuracy of preoperative ultrasonography of the contralateral groin to detect CPPV and its potential value to predict MCIH in children with unilateral inguinal hernia.

Seven studies were included in this meta-analysis. They were subdivided into two groups: ‘complete’ and ‘incomplete’ cases. Studies referred to as complete cases reported both the positive and negative index test results of contralateral exploration and showed high pooled sensitivity (93%) and moderate pooled specificity (88%). Incomplete cases reported only the positive index test results of contralateral exploration and the negative index test results of the findings of clinical follow-up, and show lower pooled sensitivity (86%) though higher pooled specificity (98%). AUC implies that diagnostic performance of preoperative ultrasonography is very good at detecting CPPV in complete (0.984) and incomplete cases (0.996). These results indicate that preoperative ultrasonography is less likely to result in a high number of false negatives in the complete cases. In the incomplete cases, the false positive rate (FPR) is low (2.1%) compared to the FPR of complete cases (11.5%). Negative preoperative ultrasonographic test results yield positive perioperative findings during contralateral exploration more often compared to the amount of clinically apparent hernias that actually develop postoperatively. These results suggest that patency of the contralateral PV is much more often detected during contralateral exploration than an MCIH actually develops during follow-up.

It is reported that at first presentation, 40% of the children diagnosed with a unilateral inguinal hernia also have a patent PV on the contralateral side [24–26]. However, the process of obliteration of a patent PV continues in the first few months of life, causing a successive decrease in the PV patency incidence and thus the incidence of positive surgical findings with advancing age. A patent PV during contralateral exploration will therefore not necessarily develop into a clinically relevant inguinal hernia: the estimated childhood risk of developing a clinically apparent inguinal hernia in case of the presence of a patent PV is only between 25% and 50% [24]; however, it considerably increases the risk for MCIH development, and risk factors to predict whether a CPPV actually develops into an MCIH or not need to be identified. This corresponds to the results of Kaneda et al., who related both the positive and negative ultrasonographic test results to clinical follow-up and reported a high ‘false positive rate’ of 75% (27/36). In contrast to the other studies included in this review, the number of true positives is extremely low (Tables 3, 4 and 5), implying that only 25% of the CPPVs actually developed into a clinically apparent MCIH. Prevalence of CPPV as determined by preoperative ultrasonography was 34.3%, though after clinical follow-up the prevalence of MCIH decreased to 10.5% [13]. As the index test results are considered positive on the basis of the presence of CPPV, regardless whether it actually develops into a clinically apparent MCIH or not, one can assume that preoperative ultrasonography overestimates MCIH prevalence (Tables 2 and 3).

The preoperative major mean CPPV axis as determined using ultrasound was significantly wider in patients with contralateral inguinal hernia compared to patients without contralateral hernia. Unfortunately, there was great variability in the diameter of the contralateral WLIR among the included studies. The mean WLIR in children diagnosed with contralateral hernia or patent PV was 2.70 ± 1.17 mm, 6.8 ± 1.3 mm or 9.0 ± 1.9 mm [13, 20], whereas in other studies children with a mean WLIR of 1.34 ± 1.26 mm, 3.5 ± 0.4 mm and less than 6.0 mm were diagnosed as having no patent PV [13, 19, 20]. Previously, Erez et al. prospectively compared the sonographic dimensions of the symptomatic, unilateral inguinal canal with the surgical findings in 642 children undergoing inguinal hernia repair and perioperatively found a PPV, ‘full hernia’ and ‘large hernia’ when a mean groin width of 4.9 ± 1.1 mm, 7.2 ± 2.0 mm and 12.8 ± 3.6 mm was ultrasonically detected [27].

One of the difficulties interpreting our study results is that the diagnostic ultrasonographic criteria to detect CPPV differ among the included studies (Table 2). First, some studies differentiate between CPPV and contralateral inguinal hernia, while others consider CPPV as potential hernia sac and include them in the category ‘contralateral hernia’ [5, 14, 23]. Thus, unlike others, Erez et al. used the hypoechoic width at the middle of the inguinal canal as the point of reference, instead of e.g. the maximum diameter or width of the inguinal canal at the internal ring [27]. A CPPV or contralateral hernia is further considered as the patency of the PV, as the presence of fluid, bowel loops or omentum in an open PV, or as the diameter of the internal ring exceeds a certain value (e.g. ≥ 4 or 4.5 mm). This shows the necessity of establishing more adequate homogeneous ultrasonographic criteria to accurately detect CPPV [28]. The results should be interpreted with caution because of high heterogeneity. Most studies included in our meta-analysis (*n* = 7) only explored the contralateral groin if preoperative ultrasonography yielded positive test results. This indicates a higher probability of the presence of CPPV during surgery, which could lead to an overestimation of the index test results. False negative rates of ultrasonography (FNR) for CPPV and FNR regarding the occurrence of MCIH might also be imprecise, since only four studies surgically explored all contralateral groins irrespective of the preoperative ultrasonographic test results [14–17] and only four studies have reported postoperative outcomes among patients who were not explored (Tables 1 and 2) [13, 18–20]. The use of different cut-off values to detect CPPV in the included studies might cause threshold effects to occur, also leading to heterogeneity of the results. Unfortunately, this meta-analysis did not provide enough data to explore the effects of differences in study characteristics on the estimates of the diagnostic test accuracy.

In conclusion, preoperative ultrasonography of the contralateral groin to detect CPPV seems promising, but may result in an overestimation of MCIH prevalence. More adequate, unequivocal ultrasonographic criteria are mandatory for proper diagnosis of CPPV and careful consideration should be given to what the appropriate reference standard is to establish accurate diagnosis of MCIH. Finally, risk factors need to be identified that can predict whether a CPPV actually develops into a clinically relevant MCIH or not.

## Electronic supplementary material

ESM 1 (DOC 36 kb)

ESM 2 (DOC 29 kb)

ESM 3 (DOC 139 kb)

## Abbreviations

CPPV: Contralateral patent processus vaginalis
MCIH: Metachronous contralateral inguinal hernia
PRISMA: Preferred Reported Items for Systematic Reviews and Meta-Analysis statement
PROSPERO: International Prospective Register of Systematic Reviews
PV: Processus vaginalis
WLIR: Width of low echoic region of the internal ring
