# Supplementary material for: Diagnostic accuracy of preoperative ultrasonography in predicting contralateral inguinal hernia in children: a systematic review and meta-analysis
Source: Eur Radiol. 2018 Jul 27;29(2):866–76. doi: 10.1007/s00330-018-5625-6 (PMC6302883; doi:10.1007/s00330-018-5625-6)
Supplement: Supplementary file 1 — (DOC 36 kb) [file 330_2018_5625_MOESM1_ESM.doc]

**SEARCH STRATEGY PUBMED**

370 hits

("Hernia, Inguinal"[Mesh] OR inguinal herni*[tiab] OR hernia inguin*[tiab] OR hernia repair*[tiab] OR pediatric herni*[tiab] OR paediatric herni*[tiab] OR groin herni*[tiab]) AND ("Infant, Newborn"[Mesh] OR "Infant"[Mesh] OR "Child"[Mesh]  OR "Child, Preschool"[Mesh] OR "Adolescent"[Mesh] OR "Pediatrics"[Mesh] OR child*[tiab] OR neonate*[tiab] OR infant*[tiab] OR newborn*[tiab] OR baby[tiab] OR babies[tiab] OR pediatric*[tiab] OR paediatric*[tiab] OR adoles*[tiab] OR teen*[tiab] OR preschool*[tiab] OR pre-school*[tiab] OR boy* [tiab] OR girl*[tiab] OR kid[tiab] OR kids[tiab] OR youth*[tiab]) AND ("Ultrasonography"[Mesh] OR ultraso*[tiab] OR sonograph*[tiab] OR echo*[tiab])

**SEARCH STRATEGY EMBASE (Ovid):**

| **#** | **Database(s): Embase Classic+Embase 1947 to 2016 December 21** | **Results (n)** |
| --- | --- | --- |
| 1 | inguinal hernia/ or patent processus vaginalis/ | 17917 |
| 2 | ((inguin* or p?ediatric or groin or repair*) adj3 herni*).ti,ab,kw. | 24252 |
| 3 | 1 or 2 | 28357 |
| 4 | child/ or preschool child/ or adolescent/ or infant/ or newborn/ or exp pediatrics/ or (child* or infan* or newborn* or neonat* or baby or babies or pediatric* or paediatric* or adoles* or teen* or puber* or schoolchild* or preschool or pre-school or boy* or girl* or kid or kids or youth*).ti,ab,kw. | 3988267 |
| 5 | exp echography/ or (ultraso* or sonograph* or echo*).ti,ab,kw. | 926161 |
| 6 | 3 and 4 and 5 | 841 |

**SEARCH STRATEGY COCHRANE LIBRARY**

33 hits

ID Search Hits

#1 MeSH descriptor: [Hernia, Inguinal] explode all trees 1095

#2 (inguin* or pediatric or paediatric or groin or repair*) near/3 herni*:ti,ab,kw (Word variations have been searched) 2235

#3 #1 or #2 2235

#4 MeSH descriptor: [Child] explode all trees 208

#5 MeSH descriptor: [Infant] explode all trees 14681

#6 MeSH descriptor: [Adolescent] explode all trees 87541

#7 MeSH descriptor: [Pediatrics] explode all trees 642

#8 child* or infan* or newborn* or neonat* or baby or babies or pediatric* or paediatric* or adoles* or teen* or puber* or schoolchild* or preschool or pre-school or boy* or girl* or kid or kids or youth*:ti,ab,kw (Word variations have been searched) 196308

#9 #4 or #5 or #6 or #7 or #8 196313

#10 MeSH descriptor: [Ultrasonography] explode all trees 9129

#11 ultraso* or sonograph* or echo*:ti,ab,kw (Word variations have been searched) 30571

#12 #10 or #11 30902

#13 #3 and #9 and #12 33
