# Supplementary material for: Diagnostic accuracy of preoperative ultrasonography in predicting contralateral inguinal hernia in children: a systematic review and meta-analysis
Source: Eur Radiol. 2018 Jul 27;29(2):866–76. doi: 10.1007/s00330-018-5625-6 (PMC6302883; doi:10.1007/s00330-018-5625-6)
Supplement: Supplementary file 2 — (DOC 29 kb) [file 330_2018_5625_MOESM2_ESM.doc]

**Appendix 2. Data collected for each included study**

- Name of first author, year of publication
- Country of origin
- Study characteristics
  - Study design
  - Study population and patient characteristics
  - Method of selection
  - Primary and secondary outcome(s)
  - Follow-up specifications
- Index test characteristics (preoperative ultrasonography of the contralateral groin)
  - Transducer specifics
  - Experience and blinding of radiologist
  - Ultrasonographic technique and examination
  - Ultrasonographic diagnostic criteria for CPPV diagnosis
- Reference test characteristics (surgical exploration or clinical follow-up)
  - Time interval between ultrasonography and reference test
  - Blinding of the operating surgeon
  - Technical features of surgical exploration
  - Perioperative diagnostic criteria for CPPV diagnosis
  - Complications
- Outcome and diagnostic accuracy test results of preoperative ultrasonography
  - Which reference standard was used?
  - Diagnostic accuracy data (sensitivity, specificity, positive and negative predictive value).
  - Diameter of the width of low echoic region of the internal ring (WLIR)
  - Area under the receiver operating characteristic curve (AUC)
  - Metachronous contralateral inguinal hernia rate
