# Supplementary material for: Diagnostic accuracy of preoperative ultrasonography in predicting contralateral inguinal hernia in children: a systematic review and meta-analysis
Source: Eur Radiol. 2018 Jul 27;29(2):866–76. doi: 10.1007/s00330-018-5625-6 (PMC6302883; doi:10.1007/s00330-018-5625-6)
Supplement: Supplementary file 3 — (DOC 139 kb) [file 330_2018_5625_MOESM3_ESM.doc]

**A1. Surgical exploration (n=12)**

No

Yes

TP

**B2. Clinical follow-up (n=7)**

**C. No clinical follow-up (n=3)**

**A2. Clinical follow-up (n=2)**

No

Yes

No

No

POSITIVE on US

= **PPV/Hernia present**

Yes

**Preoperative ultrasonography (US) of the contralateral groin (n = 14)**

FP

NEGATIVE on US

*=* **no PPV/Hernia**

TP

FP

**B1. Surgical exploration (n=4)**

Complete cases

Yes

FN

TN

FN

TN

Complete cases

**Appendix 3. Flow chart of how diagnostic test accuracy results were determined. Both surgical and clinical findings were used to calculate true positive, false positive, false negative and false positive rates.**

*In the flow chart, ‘Yes’ means that a PPV/hernia is present; ‘No’ indicates that there is no PPV/hernia present. Complete cases were referred to as surgical exploration was performed following both positive (A1) and negative (B1) index test results. All other combinations were referred to as incomplete cases.*

*Abbreviations: US: Ultrasonography, PPV= patent processus vaginalis, TP= true positive, FP= false positive, FN= false negative, FP= false positive.*
